# The +4G Site in Kozak Consensus Is Not Related to the Efficiency of Translation Initiation

**DOI:** 10.1371/journal.pone.0000188

**Published:** 2007-02-07

**Authors:** Xuhua Xia

**Affiliations:** Department of Biology, University of Ottawa, Ottawa, Canada; The Wellcome Trust Sanger Institute, United Kingdom

## Abstract

The optimal context for translation initiation in mammalian species is GCCRCCaugG (where R = purine and “aug” is the initiation codon), with the -3R and +4G being particularly important. The presence of +4G has been interpreted as necessary for efficient translation initiation. Accumulated experimental and bioinformatic evidence has suggested an alternative explanation based on amino acid constraint on the second codon, i.e., amino acid Ala or Gly are needed as the second amino acid in the nascent peptide for the cleavage of the initiator Met, and the consequent overuse of Ala and Gly codons (GCN and GGN) leads to the +4G consensus. I performed a critical test of these alternative hypotheses on +4G based on 34169 human protein-coding genes and published gene expression data. The result shows that the prevalence of +4G is not related to translation initiation. Among the five G-starting codons, only alanine codons (GCN), and glycine codons (GGN) to a much smaller extent, are overrepresented at the second codon, whereas the other three codons are not overrepresented. While highly expressed genes have more +4G than lowly expressed genes, the difference is caused by GCN and GGN codons at the second codon. These results are inconsistent with +4G being needed for efficient translation initiation, but consistent with the proposal of amino acid constraint hypothesis.

## Introduction

While translation initiation in prokaryotes is mediated by base-paring between the Shine-Dalgarno sequence at the 5-UTR on the mRNA and the anti-Shine-Dalgarno sequence at the 3′-end of the 16S rRNA [1], [2], translation initiation in eukaryotes is mediated by the Kozak consensus [3]–[6]. The optimal context for translation initiation in mammalian species is GCCRCCaugG (where R = purine), with the −3R and +4G being particularly important [3], [6]–[8]. Molecular biology textbooks abound with the implication that the −3R and +4G should be salient features of mRNA for highly expressed proteins.

The interpretation of +4G has been controversial. It has been suggested that +4G may have little to do with initiation site recognition, but is constrained by the requirement for particular type of amino acid residue at the N-terminus of the protein [9]. One piece of supporting evidence came from a detailed study of an influenza virus NS cDNA derivative [10] which showed that both +4 and +5 sites were important and changes at these sites reduced protein production. In contrast, the +6 site (the third codon position of the second codon) is less important. A simple explanation of this result is that changes at the +4 and +5 sites alter the amino acid, whereas those at the +6 site may not.

Recent studies, especially those involving the removal of the initiator methionine (Met) and myristoylation, revived the alternative explanation of amino acid constraint for the presence of +4G in protein-coding genes. First, amino-terminal modifications of nascent peptides occur in nearly all proteins in both prokaryotes and eukaryotes, and the removal of the initiator Met, which occurs soon after the amino terminus of the growing polypeptide chain emerges from the ribosome, is not only an important amino-terminal modification in itself, but also required for further amino-terminal modifications. The efficiency of removing the initiator Met depends heavily on the penultimate (the second) amino acid, with the cleavage occurring most efficiently when the penultimate amino acid is small [11]. Alanine (Ala) and glycine (Gly) happen to be the two smallest amino acids and both are coded by G-starting codons, i.e., Ala by the GCN (where N stands for any nucleotide) and Gly by the GGN codons. The need for removing the initiator Met in proteins implies the presence of many Ala and Gly at the penultimate amino acid position and consequently many +4G due to the GCN and GGN codons coding for Ala and Gly, respectively.

Another factor contributing to the prevalence of +4G, but independent of the efficiency of translation initiation, is the myristoylation process. For example, in Coxsackievirus B3, the initiation codon is flanked by both −3R and +4G, and viral mutants with a mutation from +4G to +4C is not viable [12]. This may seem to confirm what one would expect based on the necessity of the Kozak consensus for efficient translation initiation in highly expressed genes. However, it turns out that the +4G is required in Coxsackievirus B3 not because it is essential for translation initiation, but because it is needed for coding Gly (coded by GGN). The Gly at the amino terminus, after the removal of the initiator methionine, is needed to attach to a myristoyl (C_14_H_28_O_2_) fatty acid side chain, and myristoylation occurs only on a Gly residue [13]. Myristoylation may involve many proteins, and are implicated in protein subcellular relocalization [13], apoptosis [14], [15], signal transduction [16], [17], and the virulence and colonization of pathogens [12], [18]–[21]. The need for myristoylation in proteins would contribute to the presence of +4G in CDSs.

We thus have two alternative hypotheses for the presence of +4G in protein-coding genes. The conventional translation initiation hypothesis argues that the presence of +4G is necessary for highly expressed proteins, with two predictions. First, the selection favoring +4G should drive the increased usage of amino acids coded by GNN codons (e.g., Ala coded by GCN, Asp by GAY, Glu by GAR, Gly by GGN, and Val by GUN) at the penultimate amino acid site. Second, the +4G should be more prevalent in highly expressed than in lowly expressed genes. In contrast, the amino acid constraint hypothesis, based on the amino-terminal modification involving the removal of the initiator Met and myristoylation, has two different predictions. First, not all GNN codons should have increased usage, but only GCN coding Ala and GGN coding Gly should have increased usage. Second, highly expressed genes may need more efficient N-terminal processing and may consequently need more GCN and GGN codons. This may increase the frequency of +4G in highly expressed genes relative to lowly expressed genes.

## Results

### Differential use of GNN codons at penultimate site

**Figure 1:**
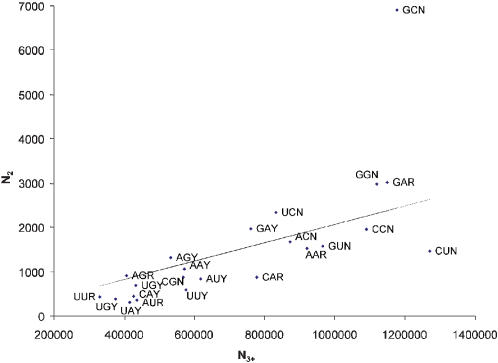
Frequencies of the penultimate codons (N_2_) relative to the frequencies of the rest of the codons (N_3+_) excluding initiation and termination codons. The line indicates the expected frequencies when the penultimate codons have the same codon usage as the rest of the codons.

Results from 34169 human coding sequences (CDSs) do not support the translation initiation hypothesis for the presence of +4G. While the five amino acids coded by GNN codons (Ala, Asp, Gly, Glu, Val) account for a majority (64.24%) of the amino acids at the penultimate site (which implies that nucleotide G is the consensus nucleotide at the +4 site), there is no consistent overuse of amino acids coded by GNN codons at the second amino acid site relative to other sites (Fig. 1). This pattern also holds for mouse genes (data not shown).

The expected number of codons (Fig. 1) at the penultimate site is calculated as follows. The total number of codons at non-penultimate sites is 16347992 (excluding the initiation and termination codon). Designate the number of codon XYZ at non-penultimate site as N_XYZ_. If codon usage at penultimate sites is the same as the rest of the genes, then the expected frequency of codon XYZ at the penultimate site is simply N_XYZ_*34169/16347992. Only alanine (GCN) codons deviate dramatically from the expected value (Fig. 1).GUN codons (coding for valine) is in fact underused at the penultimate site than at other sites (Fig. 1). Thus, there is no general increase in GNN codon usage at the penultimate site.

### Differences in +4G frequencies in highly and lowly expressed genes

The translation initiation hypothesis also predicts that highly expressed genes should be more likely to have +4G than lowly expressed genes. Ideally we should have genes with different protein expression for testing the prediction. However, there is now substantial evidence suggesting a strong correlation between mRNA level and protein production, not only in *Saccharomyces cerevisiae* [22]–[25], but also in mammalian species [26]. We used published SAGE (serial analysis of gene expression) data to characterize gene expression because comparative studies have also demonstrated a much higher reproducibility of SAGE (serial analysis of gene expression) experiments than microarray experiments in characterizing mRNA levels [27].

To check any possible differences in amino acid usage at the penultimate site and at other sites between highly and lowly expressed genes, I used the 987 unique SAGE tags that were found ubiquitously in human tissues [28]. The main reason for using ubiquitously expressed genes is that the relationship between mRNA level and protein abundance is generally weak in cell-specific genes [29].

These 987 unique tags were matched against the 34169 human CDSs. One gene (ASNA1) matched 3 tags, 16 genes matched 2 tags and 987 genes matched exactly 1 tag (The number happens to be the same as the number of unique tags, but it is accidental). For those 17 multiple-match genes (MMGs), it is difficult to assign gene expression values. For example, if a gene matches two tags, one with 10 copies/cell and another with 100 copies/cell, there is no unequivocal way of assigning an expression value to the gene. For this reason, only 987 single-match genes (SMG) are used and the data set will be referred to as SMG data set.

The SMG data set has the problem involving multiple-match tags. For example, if a tag has n copies per cell and matches two genes, say SMG1 and SMG2, it is impossible to know if the n copies/cell of the tag is contributed by SMG 1 only, or SMG 2 only, or by both. In order to assign expression values unequivocally to genes, we also compiled a more limited data set, with on 168 genes that match only single-match tags (SMTs), i.e., each gene matches only one tag which matches only one gene. These genes are designated as SSGs (to reflect the fact that they are from SMG-SMT gene-tag pairs) and their expression values range from 11 copies/cell (gene LRFN4 matching GGGGGGCUGC, excluding the leading 4-bp *Nla*III anchoring enzyme site) to 4374 copies/cell (gene GRIN2C matching GGUGACCACG). This small data set will be referred to as the SSG data set.

The 168 genes in the SSG data set were divided into a high-expression (HE) group, including 83 SSGs with expression level of at least 50 copies/cell, and a low expression (LE) group, including 85 SSGs with expression level less than 50 copies/cell. The proportion of +4G is 43.37% in the HE group and 49.40% in the LE group. We also contrasted 30 most highly expressed SSGs (expression level at least 114 copies/cell) with 30 least expressed SSGs (expression level equal to 27 copies/cell or less). The proportion of +4G is 43.33% for the former and 46.67% for the latter. Thus, there is no indication that highly expressed genes are more likely to have +4G than lowly expressed genes. The difference is in fact in the opposite direction. This result does not support the prediction of the translation initiation hypothesis.

We then categorized the 168 SSGs into five groups according to the codon at the penultimate site (GCN for alanine, GGN for glycine, GAN for aspartate and glutamate, GUN for valine, and genes without +4G designated as NonG), and compared their expression values by one-way analysis of variance (ANOVA). Gene expression differs significantly among the five groups (F = 3.07, DF1 = 4, DF2 = 163, p = 0.0180), with the average gene expression being 92.5 for GCN genes, 554.89 for GGN genes, 116.74 for GAN genes, 89.40 for GUN genes and 123.91 for NonG genes. Multiple comparisons using the LSD (least significance difference) test [30, pp. 208–209] showed that only genes with GGN (glycine) codons at their penultimate site have significantly higher expression than other groups (p<0.05). One of the genes with a glycine codon (GGU) at its penultimate site (GRIN2C matching GGUGACCACG) has a very high expression value (4374 copies/cell). Excluding this gene results in no significant difference among the five groups.

We have performed similar ANOVA for the SMG data set and found the same result, i.e., genes with GGN codons at their penultimate sites have higher expression value than the other four groups, with the average gene expression being 178.51 for GCN genes, 263.21 for GGN genes, 163.79 for GAN genes, 175.52 for GUN genes and 145.14 for NonG genes. There is no other significance difference among the five groups. Thus, we may conclude from analyzing the two SAGE data sets that (1) there is no consistent pattern that GNN codons are overused in highly expressed genes, and (2) genes with GGN (glycine) codons at their penultimate site tend to be more highly expressed than other genes. The result is inconsistent with the translation initiation hypothesis but not incompatible with the amino acid constraint hypothesis.

An alternative index of gene expression is codon adaptation index, or CAI [31], which has been shown to correlate well with published gene expression in terms of mRNA level and protein abundance [22], [23], [32]. Note that CAI is computed with a codon usage table from a set of reference genes known to be highly expressed. I used the reference set in the Ehum.cut file that is distributed with EMBOSS [33]. However, the cai program in EMBOSS is biased because it does not exclude codon families with a single codon, e.g., AUG coding methionine and UGG coding tryptophan in the standard genetic code (see Materials and Methods for details). I used DAMBE [34,35, version 4.5.10] to calculate CAI values.

We focus on two groups of genes, the high-CAI group with CAI>0.8 and the low-CAI group with CAI<0.7. Overall, high-CAI genes tend to have more genes with +4G than low-CAI genes (chi-square test, X^2^ = 25.36, DF = 1, p<0.0001, based on data in Table 1). This might seem to support the translation initiation hypothesis. However, genes with no +4G may include more false CDSs that would tend to have smaller CAI values. This would result in an association between low-CAI genes with no +4G. It is important to note that, among genes with +4G, there is little difference in their frequencies between the high-CAI and low-CAI group (Table 1, last column). The largest difference between high-CAI and low-CAI group are genes with GCN and GGN codons (coding for alanine and glycine, respectively) at their penultimate site (Table 1, last column), but the difference is not significant (p>0.05). Thus, we may conclude that only GCN and GGN codons exhibit minor differences in their frequencies at the penultimate site between high-CAI and low-CAI genes. This is again inconsistent with the translation initiation hypothesis, but somewhat compatible with the amino acid constraint hypothesis.

**Table 1:** Number of genes with GCN, GGN, GAN, GUN and non-GNN codons at their penultimate site in the high-CAI and low-CAI group (N_High-CAI_ and N_Low-CAI_), together with their proportions (P_High-CAI_ and P_Low-CAI_).

	*N_High-CAI_*	*N_Low-CAI_*	*P_High-CAI_*	*P_Low-CAI_*	*P_High-CAI_ - P_Low-CAI_*
GCN	2069	423	21.0950	19.0798	2.0152
GGN	943	162	9.6146	7.3072	2.3074
GAN	1461	309	5.9135	5.4127	0.5008
GUN	468	91	4.7716	4.1046	0.6670
NonG	4867	1232	49.6228	55.5706	−5.9478
Sum	9808	2217			

The use of CAI value [31] as an index of gene expression in human has been controversial [36], [37]. While it is well established that codon usage in bacterial species and vertebrate mitochondria is strongly constrained by the relative tRNA abundance and that there is selection pressure favoring codon-anticodon adaptation [38]–[41], there is only limited evidence for eukaryotes [42]. One additional piece of evidence supporting codon-anticodon adaptation is that the codon frequencies of the 34169 annotated human CDSs are positively correlated with the copy number of their cognate tRNA genes found at The Genomic tRNA Database (http://lowelab.ucsc.edu/GtRNAdb/), compiled with the tRNAscan-SE program [43]. For example, from the 505 human tRNA genes decoding the regular set of 20 amino acids, one can obtain their cognate codon frequencies from their anticodons. These tRNA-derived cognate codon frequencies correlate positively with the codon frequencies of the 168 genes in the SSG data set (Pearson r = 0.5731, p<0.0001, after grouping all C-ending and U-ending codons into Y-ending codons because these codons are typically translated by tRNA with nucleotide G at its wobble site). It is simpler to explain this significant positive correlation by invoking codon-anticodon adaptation than by random mutation, and suggests the utility of CAI as a measure of gene expression in human genes.

## Discussion

The original study documenting the importance of +4G [44] does not constitute a sufficient proof that +4G is important in translation initiation. The study was based on the production of proinsulin from preproinsulin. The latter has a signal peptide at its amino terminus. The signal peptide is removed during translation, generating proinsulin. When +4G is mutated to other nucleotide, the production of proinsulin is reduced. This reduced proinsulin production was assumed to be caused by reduced efficiency in translation initiation due to the mutation of +4G to other nucleotides. However, one should note that altering +4G also alter the amino acid sequence in the signal peptide and may consequently affect the removal of the signal peptide, leading to reduced production of proinsulin. Thus, the result is compatible with the amino acid constraint hypothesis for the presence of +4G.

One may think that the translation initiation hypothesis is partially correct because, after all, two of the five amino acids with G-starting codons (especially alanine) show increased usage. This is wrong. The increased usage of alanine exists not only in eukaryotes, but also in prokaryotes [45] that do not use the scanning mechanism for translation initiation and consequently do not need the +4G. The overuse of small amino acids at the penultimate amino acid site in both prokaryotes and eukaryotes is better explained by the necessity for removing the initiator Met.

While the results above are inconsistent with the predictions of the translation initiation hypothesis, they generally appear to support the amino acid constraint hypothesis. First, the latter predicted the overuse of Ala and Gly at the second codon position (to facilitate the removal of the initiator Met and myristoylation), and Ala and Gly (especially Ala) are indeed overused (Table 1). Second, all differences between highly expressed and lowly expressed genes involve GCN and GGN (coding for alanine and glycine, respectively) codons.

Both amino acid constraint hypothesis and the translation initiation hypothesis have difficulties in explain certain observations. For example, Kozak [6] found that +4G generally enhances translation initiation, but does not when it occurs in a GUN codon (coding for valine). An associated finding in this paper is that GUN is underused at the penultimate site (Fig. 1). While such findings are difficult to explain by the translation initiation hypothesis, they are also difficult for the amino acid constraint hypothesis unless valine at the penultimate site reduces the efficiency of initiator Met cleavage. Previous studies on prokaryotes and eukaryotes [46] and on the yeast, *Saccharomyces cerevisiae* [11], suggest that cleavage of initiator Met occurs with valine at the penultimate site, but a recent study on *Escherichia coli* [47] demonstrates that peptides with Val at the penultimate site dramatically reduces the efficiency of initiator Met cleavage relative to other amino acids such as Ala, Cys, Gly, Pro, or Ser in this position. Further studies on initiator Met cleavage on mammalian species are needed before one can reach a solid conclusion.

In summary, we conclude that the presence of +4G is poorly explained by the translation initiation hypothesis that claims the necessity of +4G for efficient translation initiation, but well explained by the alternative amino acid constraint hypothesis that claims the necessity of Ala and Gly at the second amino acid position in many proteins (for the removal of the initiator Met or myristoylation) as the cause of the prevalence of +4G because Ala and Gly happen to be coded by GCN and GGN codons. The necessity of +4G for efficient translation initiation appears to be a misconception that has existed in the molecular biology textbooks for too long.

## Materials and Methods

I retrieved the rna.gbk.gz file at ftp://ftp.ncbi.nih.gov/genomes/H_sapiens/RNA/, dated Sept. 3, 2006, and extracted all 34169 annotated coding sequences (CDSs) for evaluating the translation initiation hypothesis and the amino acid constraint hypothesis. CDS extraction and computation of codon adaptation index [31], as well as the analysis of codon usage at the second codon were carried out by using DAMBE (Xia 2001; Xia and Xie 2001). CDSs that are not multiples of three or not terminated with a stop codon are excluded.

Because the translation initiation hypothesis predicts that highly expressed genes should be more likely to have +4G than lowly expressed genes, we have used genes of different expression levels to check this prediction. Gene expression level is measured in two ways in this study, one by SAGE (serial analysis of gene expression) data and one by using codon adaptation index. The use of SAGE data instead of available microarray data is mainly because of the much higher reproducibility of SAGE experiments than microarray experiments [27].

SAGE data were retrieved from http://www.nature.com/ng/journal/v23/n4/extref/ng1299-387b-S1.pdf which listed, among others, 987 unique tags that are ubiquitously expressed in different human tissues, together with their abundance in copies/cell.

We searched these tags against the 34169 human CDSs for exact matches. To facilitate presentation, we define multiple-match genes (MMGs) as those CDSs each matching multiple tags, single-match genes (SMGs) as those each matching a single tag, multiple-match tags (MMTs) as those tags each matching multiple CDSs, and single-match tags (SMTs) as those each matching a single CDS. It is difficult to assess the expression level of an MMG because different tags it matches have different copies/cell values. The presence of MMTs causes an even more serious problem. For example, when a tag with 100 copies/cell matches two genes, it is impossible to know if the 100 copies are contributed by only one gene or by both. It would be methodologically wrong to assign both genes an expression value of 100 copies/cell. For this reason, we have compiled two data sets, one including all SMGs and the other including only SMGs that match SMTs (i.e., a SMG that matches a MMT is not included).

CAI for a gene is computed from (1) the codon frequencies of the gene and (2) a codon usage table from a set of reference genes known to be highly expressed, according to the following equation [31]:
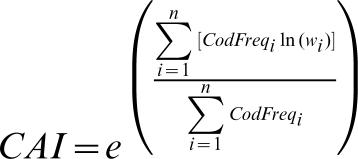
where w_i_ is computed from the Ehum.cut file distributed with EMBOSS [33] and n is the number of sense codons (excluding codon families with a single codon, e.g., AUG for methionine and UGG for tryptophan in the standard genetic code). Note that the exponent in equation (1) is simply a weighted average of ln(w). Because the maximum of w is 1, ln(w) will never be greater than 0. Consequently, the exponent will never be greater than 0. Thus, the maximum CAI value is 1.

It is important to exclude codon families with a single codon. Note that for such codons (e.g., AUG and UGG in the standard genetic code), their corresponding w_i_ value will always be 1 regardless of codon usage bias of the gene. If a gene happens to use a high proportion of methionine and tryptophan, then it will have a high CAI value even if the codon usage is not at all biased. The cai program in EMBOSS [33] does not exclude codon families with a single codon because the CAI values from that program are the same as those I computed without excluding the AUG and UGG codons. I used DAMBE [34,35, version 4.5.10] which excludes AUG and UGG in computing CAI.

The reason for using CAI as an index of gene expression instead of taking advantage of the availability of gene expression data is that, in higher eukaryotes such as human, many genes are highly expressed only at specific time and in specific tissues. For this reason, a gene with no detectable expression in a specific study, which typically involves few time points and few tissues, should not be taken as a low expressed gene. However, the availability of the gene expression data does vindicate the use of CAI as a general measure of gene expression [32].

Because CAI is based on codon frequencies of a gene with respect to the codon usage of a reference set of genes known to be highly expressed, short sequences with few codons may produce unreliable CAI values. For this reason, two separate analyses were performed, one with all CDSs and the other excluding CDSs shorter than 300 bp. The two sets of results are almost identical because short CDSs constitute only a small fraction.
